# How does Covid-19 affect global equity markets?

**DOI:** 10.1186/s40854-021-00330-5

**Published:** 2022-03-01

**Authors:** Eddie C. M. Hui, Ka Kwan Kevin Chan

**Affiliations:** ZN744, Department of Building and Real Estate, The Hong Kong Polytechnic University, Hung Hom, Hong Kong, China

**Keywords:** Covid-19, Confirmed cases, Panel regression, Equity index

## Abstract

This study applies OLS, panel regression and Granger causality test to investigate the impact of the Coronavirus disease 2019 (Covid-19) outbreak on the global equity markets during the early stage of the pandemic. We find that the Covid-19 outbreak has a significant negative impact on the overall equity index return of the eight economies even at 0.1% significance level. Furthermore, the pandemic has a more significant impact on the European countries than on the East Asian economies. The results have three main implications. Firstly, policy makers should react fast to mitigate the impact of a crisis. Secondly, investors should be aware of an outbreak of disease or other risks and adjust their investments accordingly. Furthermore, the Covid-19 outbreak results in a shift of power from the west to the east.

## Introduction

The Coronavirus disease 2019 (Covid-19) outbreak started in Wuhan, China in December 2019. The disease got widespread heading into 2020, causing over 100,000 confirmed cases of Covid-19 and over 4800 deaths in mainland China until now. The disease was most severe in China in early February, recording over 3000 confirmed cases each day. Hong Kong recorded its first confirmed case of Covid-19 on January 23 and has recorded totally about 12,000 cases until now. On 5 February 2020, Hong Kong’s Chief Executive Carrie Lam announced that starting from 8 February 2020, all people entering Hong Kong from mainland China had to undergo a mandatory quarantine for 14 days.

In mid-February, the disease gradually slowed down in China, but spread to other regions in the world. There was a Covid-19 outbreak in a church in Daegu, South Korea in mid-February. The disease spread rapidly to the whole country later. In March, the disease became even more widespread over the world. The disease began to spread rapidly in the U.S. in mid-March, too. On 11 March 2020, World Health Organization (WHO) announced that Covid-19 could be characterized as a pandemic. Starting from late 2020, several variants of the Covid-19 virus appeared. In particular, the Delta variant, which was first identified in India, is almost twice as transmissible as the original Covid-19 virus. The Delta variant caused the no. of confirmed cases of Covid-19 in many countries to rebound sharply in year 2021. Until now, there are totally over 200 million confirmed cases of Covid-19 and over 4 million deaths in the world. The U.S. is the country with the largest number of confirmed cases of Covid-19 in the world, with over 33 million confirmed cases and over 600,000 deaths.

**Fig. 1:**
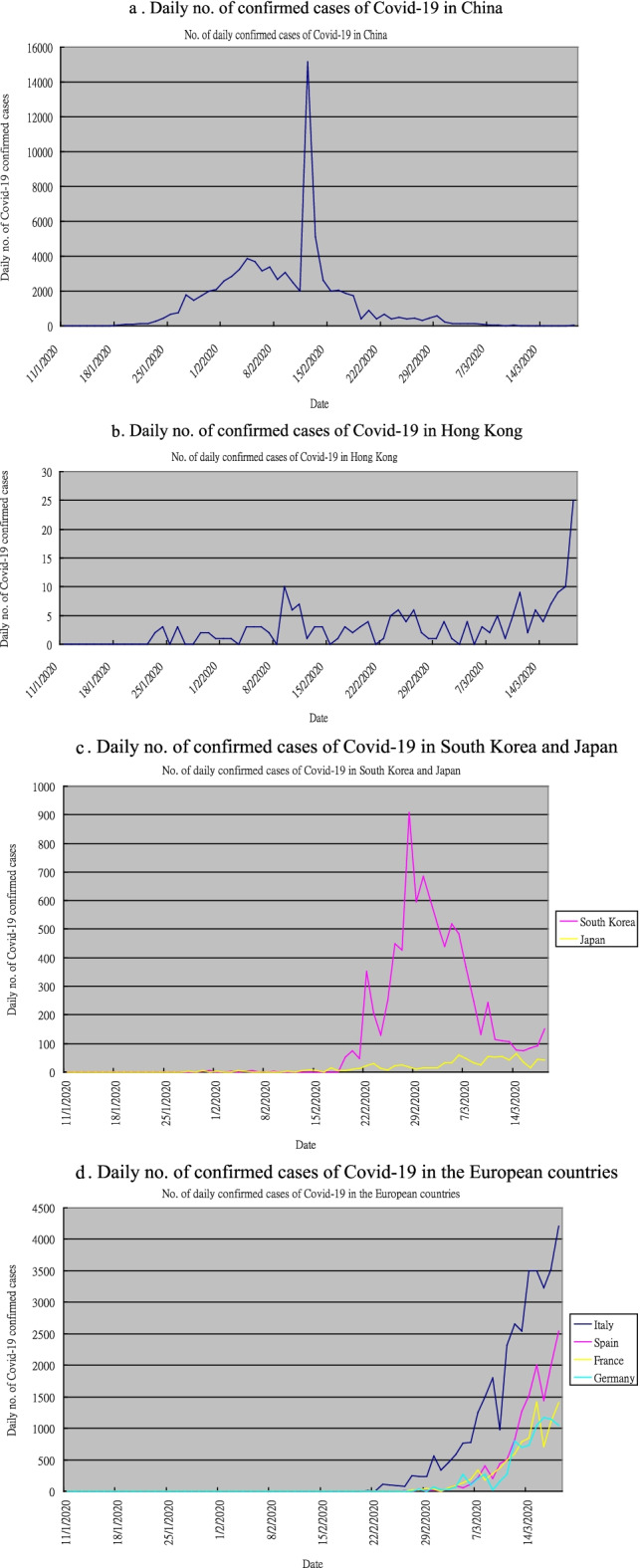
Daily no. of confirmed cases of Covid-19 in **a** China, **b** Hong Kong, **c** South Korea and Japan and **d** the European countries

Figure 1a–d display the number of confirmed cases of Covid-19 of China, Hong Kong, South Korea, Japan, Italy, Spain, France and Germany (the 8 economies selected in this study) during the period 11 January 2020–18 March 2020. We can see that the Covid-19 outbreak began in China, with the daily number of confirmed cases gradually climbing up to a peak of nearly 4000 inearly February (see Fig. 1.a). Note that the abnormally high number of confirmed cases on 12 February 2020 (about 15,000) was due to the inclusion of cases from clinical diagnosis. The number of confirmed cases decreased gradually since then. The second wave occurred in South Korea, of which the number of confirmed cases started to rise in mid-February, and reached a peak of about 900 inlate February (see Fig. 1c). The figure dropped significantly since then. The number of confirmed cases in Japan also rose starting from mid-February, but the figure was much lower than that in South Korea. The third wave occurred in Europe, with Italy being the first European country affected by Covid-19. Its number of confirmed cases of Covid-19 rose rapidly from late February onwards, reaching a peak of over 4000 on 18 March 2020 (see Fig. 1d). The figures in Spain, France and Germany climbed up in March, too. The daily number of confirmed cases of Covid-19 in Hong Kong maintained low at not more than 10 cases throughout the period, until mid-march when the influx of immigrants mainly from Europe and the U.S. caused the figure to surge to a peak of 25 on 18 March 2020 (see Fig. 1b). It seemed that Hong Kong was affected by the Covid-19 outbreak in Europe by a larger extent than the outbreak in mainland China. How did this disease affect global equity markets? According to Wagner (2020), on March 16, the Chicago Board Option Exchange’s Volatility Index (VIX), surged past the prior all-time peak. On the same day, the second-worst day ever of the Dow Jones Industrial Index occurred. This reflects that the U.S. financial market became extremely volatile during the Covid-19 outbreak. The economic growth of the U.S. also plunged to − 31.2% in Q2 of year 2020, which was the lowest figure in decades. Yet, previous studies on how the Covid-19 outbreak affects global equity markets produced mixed results. Therefore, we are still not sure what the relationship between the pandemic and the return of global equity markets is. Is the impact of the pandemic on global equity markets significant? What are the regions most significantly affected by the pandemic? This is the motivation of our study, which aims to investigate the above two questions. This is important as investors have become more concerned about stock price crash risk. Wen et al. (2019) investigate the effect of retail investor attention on stock price crash risk in China. Their findings not only enrich the extant literature on the determinants of stock price crash risk, but also give some suggestions for investors in the emerging market. The main contribution of our study is that we investigate how the Covid-19 outbreak affected global equity markets in two ways: (1) on each individual economy, and (2) globally as a whole. The results would be useful for investors to reallocate their investment and for policy makers to implement appropriate policies when an outbreak of a disease or other crises occurs.

Severe Acute Respiratory Syndrome (SARS) is the first new, serious and contagious illness of the twenty-first century. Like Covid-19, SARS was also caused by coronavirus. We can learn lessons from the SARS outbreak to explain events in the Covid-19 outbreak. SARS was originated in China in November 2002 and spread to Hong Kong in February 2003. Other parts of the world also reported cases of SARS contraction, too. The SARS outbreak ended in July 2003. A total of 8096 people contracted SARS and 774 people died of the disease in the world. Over 65% of the cases were reported in China, with 5327 people contracting SARS and 349 deaths, followed by Hong Kong, with 1755 confirmed cases and 299 deaths. SARS caused an economic downturn in both China and Hong Kong. In Hong Kong, SARS also caused a fall in housing price. The Centa-city Leading (CCL) Index plunged from 35.66 in early February to a trough of 32.11 in mid July, which was a 10% fall.

Alfaro et al. (2020) model cumulative SARS and Covid-19 infections as either exponential or logistic, and re-estimate the parameters of these models each day of the outbreak using information reported up to that day. They apply panel regression to find that a doubling of predicted infections is associated with a 4–11% decline in aggregate market value. This result implies a decline in returns' volatility as the trajectory of the pandemic becomes clearer. Our model is somewhat similar to Alfaro et al. (2020)’s model. However, they use predicted number of cases of infection in their regression model. Instead, this study uses the actual number of cases of infection to reflect the actual situation. Furthermore, the study of Alfaro et al. (2020) is firm-level. They use various US stocks in their model for Covid-19. In comparison, this study is market-level and aims to analyze the impact of the Covid-19 outbreak on the global financial market. The timeline of this study is the early period of the Covid-19 outbreak: 11 January 2020–18 March 2020. We select the following eight economies: China, Hong Kong, South Korea, Japan, Italy, Spain, France and Germany. For each economy, one equity index is chosen. We apply panel regression to examine how the number of confirmed cases of Covid-19 affects the index return as a whole. Besides testing the overall effect by panel regression, we also set up OLS regression models to investigate the impact of the Covid-19 outbreak on the index return of each individual economy. In addition, we add the following new feature which does not appear in any of previous studies on Covid-19 and SARS: We conduct Granger causality test to examine the causality relationship between:

1. the number of confirmed cases of Covid-19 of different economies,
2. the equity index return of different economies, and
3. the number of confirmed cases of Covid-19 and equity index return of each economy.

An advantage of Granger causality test is that it can determine whether a time series is useful in forecasting another. Thus we can identify the direction of transmission of shocks between the economies. We can see that which economies are sources and recipients of shocks. This information is useful for investors.

Note that there are other econometric methods that analyze the co-movements or contagion effects between financial markets. For example, Kenourgios et al. (2019) apply a dynamic conditional correlation analysis and several robustness tests to examine the effects of the unconventional monetary policy (UMP) launched by the European Central Bank on the cross-market correlations between bond, stock and currency forward markets. They find a spillover effect on both developed and emerging markets, although this impact is not identical across assets and countries. Furthermore, the new UMP phase which began in 2014 has a more prominent impact.

The paper continues as follows: “Literature review” section summarizes previous literature on related topics. “Data and model” section describes the data source and sets up the model of panel regression. “Result and analysis” section displays and analyzes the results. Finally, we draw up conclusions “Discussion and conclusion” section.

## Literature review

There are a number of studies on the impact of the Covid-19 outbreak on equity or real estate markets. Contessi and De Pace (2021) identify periods of mildly explosive dynamics and collapses in the stock markets of 18 major countries during the first wave of the COVID-19 pandemic. They find statistical evidence of instability transmission from China to all other markets. Al-Awadhi et al. (2020) use panel data analysis to test the effect of the Covid-19 outbreak on the stock market of China. They find that both the daily growth in total confirmed cases and in total death cases caused by Covid-19 have significant negative effects on stock returns across all companies. Mazur et al. (2021) examine the performance of the U.S. stock market during the first wave of the Covid-19 outbreak in March 2020. They find that natural gas, food, healthcare, and software stocks earn high positive returns, while equity values in petroleum, real estate, entertainment, and hospitality sectors fall dramatically. Furthermore, underperforming stocks exhibit extreme asymmetric volatility that correlates negatively with stock returns. Gao et al. (2021) apply a novel wavelet-based quantile-on-quantile method to compare the impact of COVID-19 on stock market volatility between the U.S. and China. They find that COVID-19 is the main reason for the sharp fluctuation of the U.S. stock market. However, unlike China, the strong growth of daily new cases, which continues for months, has made the U.S. stock market insensitive to COVID-19. In addition, the loose interest rate policy has effectively suppressed the volatility of the U.S. stock market. The result shows the differences of the financial market response under different epidemic management modes. This is of great practical significance towards achieving epidemic control and financial market stability under the background of the global spread of COVID-19. Baker et al. (2020) use text-based methods to investigate the unprecedented stock market reaction to the Covid-19 outbreak with respect to large daily stock market moves back to 1900 and with respect to overall stock market volatility back to 1985. They argue that policy responses to the Covid-19 outbreak provide the most compelling explanation for its unprecedented stock market impact. Ramelli and Wagner (2020) analyze the stock price effects of the Covid-19 outbreak. They find that the telecom and health care industries do relatively well, while transportation and energy plummet. Within industries, U.S. firms reliant on Chinese inputs and those with a strong export orientation towards China suffer. Gormsen and Koijen (2020) use data from the aggregate equity market and dividend futures to quantify how investors’ expectations about economic growth across horizons evolve in response to the coronavirus outbreak. As of March 18, their forecast of annual growth in dividends is − 28% in the US and − 25% in the EU, and their forecast of GDP growth is − 2.6% both in the US and in the EU. The lower bound on the change in expected dividends is − 43% in the US and − 50% in the EU on the 3-year horizon. Alfaro et al. (2020) examine how predicted infections during the SARS and Covid-19 outbreaks forecast aggregate equity market returns by panel regression. The results imply that a doubling of such predictions is associated with a 4–11% decline in aggregate market value, which indicates a decline in returns' volatility as the trajectory of the pandemic becomes clearer. De Vito and Gomez (2020) investigate how the Covid-19 outbreak affects the liquidity of listed firms across 26 countries. They stress-test three liquidity ratios for each firm with full and partial operating flexibility in two simulated distress scenarios corresponding to drops in sales of 50% and 75% respectively. Applying SutteARIMA method, Ahmar and Val (2020) predict the short-term of confirmed cases of covid-19 and IBEX in Spain. They find that the MAPE value is 0.036 for confirmed cases of Covid-19 in Spain and is in the amount of 0.026 for IBEX stock. Arif et al. (2021) present the time–frequency connectedness between green and conventional financial markets. In particular, they perform a subsample analysis for the COVID-19 pandemic crisis period. They observe an enhanced connectedness during the COVID-19 period, suggesting that financial stability is a significant factor in determining the smooth transition to green investments. Li et al. (2021a, b) explore the dynamics of the return connectedness among major commodity assets and financial assets in China and the US during the COVID-19 pandemic by using the time-varying connectedness measurement. They find that the total return connectedness of the US commodity and financial assets is stronger than that of the Chinese commodity and financial assets in most cases. The interchangeable roles of the commodity and financial assets suggest flexible regulatory and portfolio allocation strategies should be applied by policy makers and investors. Umar et al. (2020) investigate the connectedness between major environmental, social, and governance (ESG) leader equity indices. They find that developed markets are the shock transmitters to Asian and other emerging markets. In particular, the COVID-19 pandemic boosts further the connectedness among the markets. Gubareva and Umar (2020) apply wavelet analyses to study the impact of COVID-19 pandemic on the performance of emerging market bonds, in both investment grade and high yield ranges of creditworthiness. They find varying level of coherence ranging from low, medium and high between the Coronavirus Media Coverage index and the price moves of the emerging market USD-denominated debt. Farid et al. (2021) investigate volatility connectedness across precious metals, energy and US stocks before and during the COVID-19 outbreak. They find significant impact of the COVID-19 pandemic on the volatility linkages of financial markets as the volatility connectedness among the different assets peaked during the outbreak. Papadamou et al. (2021) investigate the impact of the recent COVID-19 pandemic on the time-varying correlation between stock and bond returns of 10 countries. They employ both a panel data specification and a wavelet analysis to identify flight-to-quality episodes. Their results show that global stock and bond markets offer diversification to investors during the COVID-19 crisis. Milcheva (2021) uses the global systemic shock associated with the Covid-19 outbreak to assess the risk-return relationship in the cross-section of real estate equities in the US and in selected Asian countries. She finds that during the early stages of the pandemic, the sensitivity of Asian real estate companies to the market becomes negative, while it remains positive and increases in the US. Real estate sectors experience strong divergence in performance in the US while little sectoral difference is observed in Asia. Harjoto et al. (2021) find that COVID-19 causes a negative shock to the global stock markets, especially in emerging markets and for small firms.

There are some studies on the impact of SARS on equity and real estate markets. Wong (2008) uses a panel data set of large-scale housing complexes (estates) to investigate the cross-sectional variation in the spread of SARS to estimate the impact of the disease on real estate prices and sales. She finds that the average housing price declines by 1–3% if an estate is directly affected by SARS, and by 1.6 percent for all estates as a result of the SARS outbreak. The low figure contrasts with the predictions of overreaction from psychological and behavioral economics theories. This is likely to be related to housing market characteristics, including transaction costs, credit constraints and loss aversion by an analysis of transaction volume. Chen et al. (2018) examine the effect of the SARS epidemic on the long-run relationship between China and four Asian stock markets. They find the existence of a time-varying cointegration relation in the aggregate stock price indices. They also find that the SARS epidemic weakens the long-run relationship between China and the four markets. Wu (2003) tests the impact of the SARS epidemic on the closed-end and the open-end fund industries in China. The result shows that the impact is limited and transitory. Compared with the closed-end fund, the open-end fund realizes positive abnormal returns earlier at the end of the event-window period, implying that this industry recovered faster. Loh (2006) examines the impact of SARS on the performance and risk profiles of a set of airline stocks listed at the stock markets of Canada, China, Hong Kong, Singapore and Thailand. He find that airline stocks are more sensitive to news about SARS compared with the average non-aviation stock, and the negative repercussions of SARS on stocks surfaces in the form of increased volatilities rather than lower mean returns. He also finds that airline stocks tend to adopt an "aggressive" nature in the wake of SARS. Siu and Wong (2004) examine the economic impact of SARS on Hong Kong. They find that the most significant negative effects are on the demand side, with local consumption and the export of services related to tourism and air travel severely affected in the short run. In particular, they examine the trend of major global equity indices during the SARS outbreak. Out of the four sub-indices of Hang Seng Index in Hong Kong, Hang Seng Property Index records the largest percentage fall during the periods 12 March 2003 – 31 March 2003 (− 4.68%) and 31 Match 2003–15 April 2003 (− 4.62%). This reflects that the property sector is the sector which is most severely hit by the SARS outbreak in Hong Kong. Bucchianeri (2010) investigates the link between various risk factors, including socioeconomic status (SES), and the spread of SARS in Hong Kong in 2003. She finds a negative and significant correlation between the SARS outbreak and various measures of income, but not years of education. This result contrasts with previous studies on other health conditions. The income-SARS gradient can be accounted for by controlling for pre-SARS housing values but not an array of measurable living conditions. Wu et al. (2017) apply a Bayesian Vector-Autoregressive (BVAR) model with sign restrictions to estimate the underlying drivers of Hong Kong’s housing price dynamics during the period 1996–2016. They find that shocks to bank lending, housing supply, and housing demand are the main contributors to the fall in housing price during the Asian financial crisis–SARS episode. The contribution of housing demand shock has become more prominent in the down-cycle after 2000, including the SARS outbreak, showing that the SARS epidemic leads to a significant fall in housing demand. Argyroudis and Siokis (2019) investigate the impact of the sub-prime loan crisis on the Real Estate Market of Hong-Kong. They apply permutation entropy, complexity–entropy causality plane and Tsallis complexity–entropy curve to characterize the complexity of the housing indices in terms of both size and region, and distinguish the level of informational efficiency. They identify periods where the underlying dynamical structure of the market was impacted by certain events like the SARS epidemic. Their two permutation entropy approaches seem to capture well the SARS epidemic in 2003. After the SARS outbreak, all indices decline in terms of permutation entropy value for more than 2 years.

From the above, there are a number of studies on the impact of the Covid-19 outbreak on the equity or real estate market. However, Covid-19 is a new virus and the pandemic is still ongoing. Hence the studies produce mixed results. Some studies are at firm-level, while some are at market-level. The majority of the studies show that the pandemic has a significant negative impact on stock prices or stock indices. Some studies apply regional analysis to investigate the impact of the pandemic on different regions. However, what is the direction of transmission of shocks? There seems to be no absolute answer. We can see that existing literature has only marginal contribution. In order to fill in this gap, this study contributes to the literature by investigating the direction of transmission of shocks between the no. of confirmed cases of Covid-19/index return of different economies, and between the two time series of the same economy.

## Data and model

East Asia and Europe are the two regions mainly affected by early stage of the Covid-19 outbreak, so we select four economies each from East Asia and Europe. China is the country which recorded the first confirmed case of Covid-19 in the world. South Korea, of which the Covid-19 outbreak already began in February, was the East Asian country with the second largest number of confirmed cases of Covid-19 as of 18 March 2020. Hong Kong and Japan rank among top 3 in market cap among all Asian stock exchanges, and they recorded significant number confirmed cases of Covid-19 in early 2020, too. For Europe, we choose the four countries with the largest number of confirmed cases of Covid-19 until 18 March 2020: Italy, Spain, France and Germany. They are also the major economies in Europe.

For each of the eight economies, we select an equity index which consist of the most frequently traded equities in the corresponding economies, and are widely accepted as benchmarks. We obtain data of the following equity indices from Bloomberg:

China: Shanghai Composite Index

Hong Kong: Hang Seng Index

South Korea: Korea Composite Stock Price Index

Japan: Nikkei 225 Index

Italy: Milan Stock Exchange MIB 30 Index

Spain: Madrid Stock Exchange General Index

France: CAC Index

Germany: DAX Index

Next, we obtain the daily number of confirmed cases of Covid-19 in each economy. Compared with Alfaro et al. (2020) who use the predicted number of cases of infection, this study uses the actual number of cases of Covid-19 infection to reflect the actual situation truly. The daily number of confirmed cases of Covid-19 in China is obtained from the website of National Health Commission of the People’s Republic of China (National Health Commission of the People’s Republic of China 2020) (the data is not available on or before 10 January 2020), while the daily number of confirmed cases of Covid-19 in other countries is obtained from the website of Centre of Health Protection in Hong Kong (Centre of Health Protection 2020). Note that we exclude cases recorded on the Diamond Princess Cruise from the daily number of confirmed cases of Covid-19 in Japan.

Then we select the period of observation. The number of confirmed cases of Covid-19 on each day is recorded from 00:00 to 24:00 on that day, which is beyond the closing time of all selected stock exchanges. Therefore, we assume that for a particular economy, the number of confirmed cases of Covid-19 on each day would affect the index return of that economy on the following day. Since all stock exchanges trade from Monday to Friday only, and we assume that for each economy, the no. of confirmed cases on day $t$ would affect the index return on day $t + 1$, we only include data of number of confirmed cases of Covid-19 from Sunday to Thursday when performing individual linear regression, panel regression and Granger causality test between no. of confirmed cases and index return of each economy. We take the daily number of confirmed cases of Covid-19 in the 8 economies during the period 11 January 2020–18 March 2020, the early period of the Covid-19 outbreak. Note that the daily number of confirmed cases of Covid-19 in China from 12 February 2020 to 14 February 2020 included those cases confirmed from clinical diagnosis, making the number of confirmed cases much larger than usual, so we exclude these days (14 February 2020 is excluded anyway because it is Friday). We need data of equity index return during the period 13 January 2020–19 March 2020, so we obtain data of the 8 equity indices during the period 10 January 2020–19 March 2020 (13 January 2020 is Monday. The previous trading day is 10 January 2020, which is Friday). For each day during the period 13 January 2020–19 March 2020, we calculate the continuously compounded daily return of each index as follows:

1 $$R_{i,t} = \ln \left( {\frac{{S_{i,t} }}{{S_{i,t - 1} }}} \right),$$

where $S_{i,t}$ is the equity index of economy $i$ on day $t$.

However, there are different non-trading weekdays for the 8 economies as follows:

China: 24 January 2020–31 January 2020.

Hong Kong: 27 January 2020, 28 January 2020.

South Korea: 24 January 2020, 27 January 2020.

Japan: 13 January 2020, 11 February 2020, 24 February 2020.

Hence we have to exclude the data of index return of all 8 indices on the above days (the data of daily number of confirmed cases of Covid-19 on the day before each of the above days hence should be excluded), as well as 13 February 2020 and 14 February 2020 because we exclude the data of daily number of confirmed cases of Covid-19 in 8 economies on 12 February 2020 and 13 February 2020.

Next we set up the panel regression model. Looking at the daily number of confirmed cases of Covid-19 in each economy, we can see that small numbers are most frequent, while large numbers occur rarely, so it is better to take the logarithm of the daily number of confirmed cases of Covid-19 in each economy when performing panel regression. However, we cannot take logarithm of zero, so we add one to the daily number of confirmed cases of Covid-19 in each economy before taking logarithm.

For each of the eight economies, we can set up a simple linear regression model as follows:

2 $$R_{{t + {1}}} = a + b\ln \left( {X_{t} + 1} \right) + \varepsilon_{t} ,$$

where $R_{t}$ is the continuously compounded daily return of the equity index of that economy on day $t$, and $X_{t}$ is the daily number of confirmed cases of Covid-19 in that economy on day $t$. Model (2) can test the effect of the Covid-19 outbreak on the equity index return of each individual economy.

The natural logarithm of daily cases or growth of daily cases is widely used in the literature. For example, Alfaro et al. (2020) adopt the logarithm of growth of daily cases in their model.

For a more comprehensive analysis of the overall impact of the Covid-19 outbreak on the equity index return, we set up the following fixed-effects panel regression model:

3 $$R_{{i,t + {1}}} = \alpha + \beta \ln \left( {X_{i,t} + 1} \right) + \lambda_{i} + \varepsilon_{i,t} ,$$

where $R_{i,t}$ is the continuously compounded daily return of the equity index of economy $i$ on day $t$, and $X_{i,t}$ is the daily number of confirmed cases of Covid-19 in economy $i$ on day $t$. The slope coefficient $\beta$ indicates how the index return of an economy is affected by the number of confirmed cases of Covid-19 in that economy.

## Result and analysis

We first display the descriptive statistics of $\ln \left( {X_{i,t} + 1} \right)$ and $R_{i,t}$ of the 8 economies, which are shown in the following tables:

Figure 1a–d show that the number of confirmed cases of Covid-19 of all 8 economies is highly skewed to the right. However, from Table 1a, when taking logarithm after adding one to the number of confirmed cases, we can see that the mean and median of $\ln \left( {X_{i,t} + 1} \right)$ of China, Hong Kong and Japan are close together, implicating that the data is much less skewed. The mean of $\ln \left( {X_{i,t} + 1} \right)$ of South Korea is much greater than its median, so it is still highly skewed to the right. The number of confirmed cases of Covid-19 in the four European countries remains zero for over half of the period, so the median of $\ln \left( {X_{i,t} + 1} \right)$ is zero for the four European countries. In overall, $\ln \left( {X_{i,t} + 1} \right)$ is more suitable than $X_{i,t}$ in performing regression analysis and Granger causality test.

**Table 1:** Descriptive statistics of (a) $\ln \left( {X_{i,t} + 1} \right)$ and (b) $R_{i,t}$ of the 8 economies

Economy	China	Hong Kong	South Korea	Japan	Italy	Spain	France	Germany
(a)								
Mean	2.347	0.432	1.097	0.752	1.077	0.718	0.748	0.731
Standard deviation	1.053	0.358	1.111	0.646	1.434	1.152	1.110	1.067
Median	2.605	0.477	0.540	0.739	0	0	0	0
(b)								
Mean	− 0.00190	− 0.00242	− 0.00446	− 0.00373	− 0.00155	− 0.00403	− 0.00447	− 0.00452
Standard deviation	0.00964	0.00737	0.01160	0.00785	0.01126	0.01475	0.01317	0.01253
Median	− 0.00066	− 0.00126	− 0.00128	− 0.00235	0.00208	− 0.00116	− 0.00176	− 0.00072

Table 1b shows the descriptive statistics of $R_{i,t}$, i.e. the continuously compounded daily index return of the 8 economies. The average continuously compounded daily return of all 8 equity indices is negative, showing that the Covid-19 outbreak has a negative impact on the equity markets of all 8 economies. In overall, the standard deviation of $R_{i,t}$ of the four European countries is greater than that of the four East Asian economies. The median of $R_{i,t}$ is greater than its mean for all 8 economies, showing that the continuously compounded daily return of all 8 equity indices are skewed to the left. This is due to the Covid-19 outbreak causing panic in the equity markets, leading to extremely negative index return on certain days.

We perform OLS regression to the model (2) for each of the eight economies. The results are shown in the following table:

From Table 2, the slope coefficient $b$ is positive for China only, but is negative for all other economies. This result is expected because the Covid-19 outbreak caused much fewer people to go outdoors for consumption, hitting the economy hard. The equity indices of the countries reflected the economies of those countries and hence fell. Moreover, at 5% significance level, $b$ is significant for all European countries and Japan, but is insignificant for the other three East Asian economies, reflecting that the Covid-19 outbreak has a more significant impact on the index return of the European economies. The main reason is that the East Asian economies already have experience of dealing with the SARS outbreak in 2003. They took quarantine measures at once. For example, the entire city of Wuhan was locked down on 23 January, 2020. Similar quarantine measures were implemented in other regions of China thereafter. In particular, in Hong Kong, the experience of the SARS outbreak in 2003, the prominent role of the civil society and self-discipline of citizens contributed to the low infection rate (Wan et al. 2020) Therefore, the outbreak was controlled at a limited scale. However, the European economies lack experience of dealing with such a widespread disease. The quarantine measures were implemented a bit too late. Furthermore, most East Asian citizens did not mind that their freedom may be restricted by strict controls to stop disease spreading. They tended to follow those controls. On the other hand, most western citizens believed that their freedom is more important. They thought that the preventive measures would restrict their freedom, so they were opposed to the measures. Hence the disease became out of control, hitting the European economy hard. This led to a more significant downturn in the European equity indices. A major implication of this result is that some crises are regional. For example, the Covid-19 outbreak affected the western economies more severe than the East Asian economies, so investors and fund managers should avoid equities of western economies during the outbreak. Another example is the Brexit crisis in 2016, of which Hui and Chan (2021) find that the crisis has a more significant impact on the western economies and on the Asian economies. Since the market changes quickly, investors and fund managers should take action as soon as possible, preferably at the beginning of the outbreak, in order to limit their losses. Of course there would be a time lag: the outbreak starts, the stock price falls, and then investors sell their stocks. However, the sooner you sell the stocks, the smaller the loss you incur. This is a short-term trading strategy. Only insiders may anticipate the outbreak of a crisis and take action before the crisis occurs. Shen et al. (2021) find that REIT insiders reduced their holdings significantly during the real estate boom period as early as 2004, before the global financial crisis in 2008. This result supports the informed trader hypothesis that managers and employees in REITs anticipated the burst of the real estate bubble and the imminent financial crisis, and shifted their wealth away from the real estate market to avoid potential losses.

**Table 2:** Results of the OLS regression model (2)

Economy	China	Hong Kong	South Korea	Japan	Italy	Spain	France	Germany
$b$	0.0012	− 0.0050	− 0.0034	− 0.0061	− 0.0025	− 0.0041	− 0.0048	− 0.0045
t-statistics	0.7957	− 1.6130	− 2.0008	− 3.5141	− 2.0749	− 2.1728	− 2.8406	− 2.6601
*p* value	0.4314	0.1155	0.0530	0.0012	0.0452	0.0365	0.0074	0.0116

Next, we apply the software Minitab 19 to perform panel regression to the fixed effects model (3). The results are shown in the following table:

From Table 3, $\beta$ is negative and highly significant (significant at even 0.1% level), showing that the number of confirmed cases of Covid-19 in the economies has a significantly negative impact on the return of the equity indices of the economies. A negative coefficient is expected because the Covid-19 outbreak caused much fewer people to go outdoors for consumption, hitting the economy hard. The equity indices of the countries reflected the economies of those countries and hence fell. The highly significant coefficient reflects that the impact of the Covid-19 outbreak is significant and widespread. Compared with the SARS outbreak in 2003, the Covid-19 outbreak is more widespread. There were only totally 8096 confirmed cases of SARS in 2003, with 7082 of them recorded in mainland China and Hong Kong. However, as of 29 March 2020, there were already totally over 670,000 confirmed cases of Covid-19 and this figure kept on rising to over 200 million now. Furthermore, unlike SARS which was concentrated in mainland China and Hong Kong, many regions in the world recorded significant number of confirmed cases of Covid-19. As of March 29 2020, a total of 205 countries recorded confirmed cases of Covid-19. Now almost all countries in the world record confirmed cases of Covid-19. In contrast, only 30 countries recorded confirmed cases of SARS in 2003. This shows that the Covid-19 outbreak is more widespread than the SARS outbreak in 2003. We had never faced such a widespread disease before. The Covid-19 outbreak triggered huge panic in the global financial market, so the global equity indices plunged sharply. A major implication of this result is that fund managers and investors should be aware of an outbreak of disease or other risks and adjust their investments accordingly. Another implication is that when policy makers face a crisis that may potentially affect their economy, they should react fast to mitigate the impact of the crisis. However, many governments face a dilemma between restricting the transmission risk of the virus by social distancing and maintaining economic activities. Li et al. (2021a, b) investigates the role of institutions in helping governments strike the balance between saving the lives and recovering the economy in 28 provinces in China. They highlight the importance of economic considerations in fighting COVID-19 and the roles of institutions in explaining the variations of government response strategies.

**Table 3:** Results of the fixed effects panel regression model (3)

$\beta$	Standard error	t-statistics	*p* value	$R^{2}$
− 0.003276	0.000585	− 5.60	< 0.001	0.0107

Then we conduct Granger causality test through the website Wessa (2020) to examine the causality relationship between:

1. the number of confirmed cases of Covid-19 of different economies,
2. the equity index return of different economies, and
3. the number of confirmed cases of Covid-19 and equity index return of each economy.

The results are shown in the following tables:

Table 4a shows the p-values of the Granger causality test on $\ln \left( {X_{i,t} + 1} \right)$ of the 8 economies. From the table, we can identify which economies are the major sources or recipients of the Covid-19 outbreak. At 5% significance level, the number of confirmed cases of Covid-19 in Italy significantly Granger causes the number of confirmed cases of Covid-19 in 5 other economies, so we can identify Italy as a source of the Covid-19 outbreak. The Covid-19 outbreak in Italy began in late February in the northern region and spread to other European countries and even other parts of the world. The result coincides with the actual situation. At 5% significance level, the number of confirmed cases of Covid-19 in Hong Kong is significantly Granger caused by 5 other economies: Japan and all 4 European countries. This coincides with the actual situation that the number of confirmed cases of Covid-19 in Hong Kong increased sharply in mid-March due to the large influx of immigrants and tourists mainly from Europe. There were also a number of confirmed cases imported from Japan, too. Hong Kong is clearly a recipient of the covid-19 outbreak.

**Table 4:** *p* values of the Granger causality test on (a) $\ln \left( {X_{i,t} + 1} \right)$, (b) $R_{i,t}$ of the 8 economies and (c) *p* values of the Granger causality test between $\ln \left( {X_{i,t} + 1} \right)$ and $R_{i,t}$ of each of the 8 economies

	Recipient							
(a)								
Source	China	0.698021	0.667601	0.866272	0.826572	0.572981	0.563564	0.511255
	0.263977	Hong Kong	0.560073	0.768454	0.189710	0.440045	0.530950	0.701268
	0.012415	0.058785	South Korea	0.000268	3.13 × 10^–5^	0.066047	0.057474	0.029470
	0.006643	0.007571	0.028479	Japan	0.001741	0.057504	0.015034	0.071302
	0.012658	0.007092	0.410447	0.00349	Italy	0.000460	8.85 × 10^–5^	4.30 × 10^–6^
	0.020633	0.001904	0.729311	0.030531	0.316916	Spain	0.002139	5.50 × 10^–7^
	0.020569	0.002761	0.759329	0.054139	0.382101	0.370744	France	1.31 × 10^–8^
	0.057389	0.008927	0.606892	0.086722	0.197307	0.115558	0.000476	Germany
(b)								
Source	China	0.544424	0.039571	0.838035	0.492295	0.958960	0.857592	0.879781
	0.791993	Hong Kong	0.001358	0.823920	0.913958	0.605238	0.909366	0.931905
	0.280445	0.000121	South Korea	0.169997	0.678331	0.058796	0.050618	0.028812
	0.182089	0.001932	0.231562	Japan	0.905928	0.008052	0.027710	0.017987
	0.492295	0.287735	0.471281	0.033015	Italy	0.000802	0.014574	0.011477
	0.802468	0.932675	0.853021	0.000586	0.516475	Spain	0.104732	0.136393
	0.591066	0.271251	0.715945	0.000698	0.337280	0.158152	France	0.846873
	0.556160	0.229315	0.731679	0.000549	0.415052	0.235021	0.722761	Germany
(c)								
	China	Hong Kong	South Korea	Japan	Italy	Spain	France	Germany
$\ln \left( {X_{i,t} + 1} \right)$ -> $R_{i,t}$	0.121281	0.437926	0.253122	0.057691	0.209052	0.00234	0.000676	0.002094
$R_{i,t}$ -> $\ln \left( {X_{i,t} + 1} \right)$	0.794184	0.294549	0.651025	0.446594	0.648026	0.758888	0.487102	0.420531

However, there is an abnormal result. At 5% significance level, the number of confirmed cases of Covid-19 in China is significantly Granger caused by 5 other economies: South Korea, Japan, Italy, France and Germany. In order to find out the reason for this abnormal result, we first calculate the correlation between $\ln \left( {X_{i,t} + 1} \right)$ of China and other 7 economies:

From Table 5, $\ln \left( {X_{i,t} + 1} \right)$ of China is weakly positively correlated to $\ln \left( {X_{i,t} + 1} \right)$ of Hong Kong only, but is negatively correlated to other 7 economies. In particular, the magnitude is larger for the correlation between $\ln \left( {X_{i,t} + 1} \right)$ of China and the 4 European countries. Hence we can see that the no. of confirmed cases of Covid-19 in China is negatively correlated to other economies except Hong Kong. The negative correlation is stronger with the 4 European countries.

**Table 5:** Correlation between $\ln \left( {X_{i,t} + 1} \right)$ of China and other 7 economies

Economies	Hong Kong	South Korea	Japan	Italy	Spain	France	Germany
Correlation	0.113084	− 0.180256	− 0.183503	− 0.399242	− 0.442141	− 0.415787	− 0.404937

**Fig. 2:**
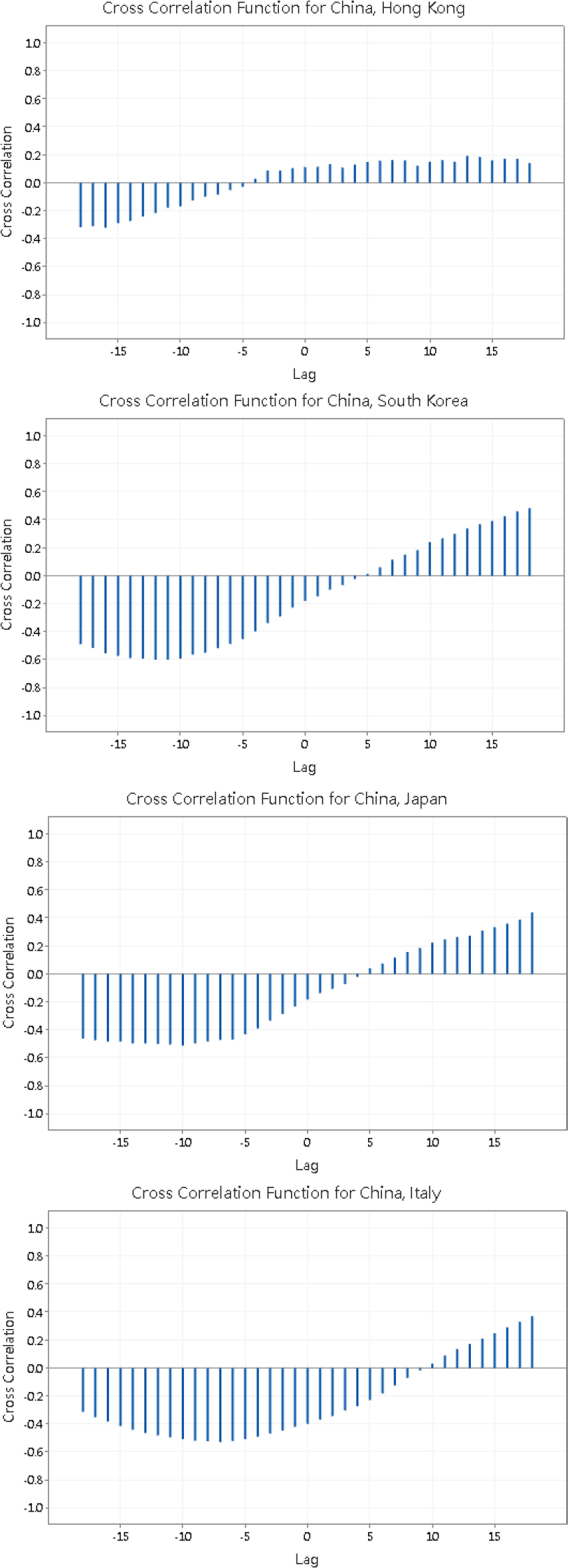

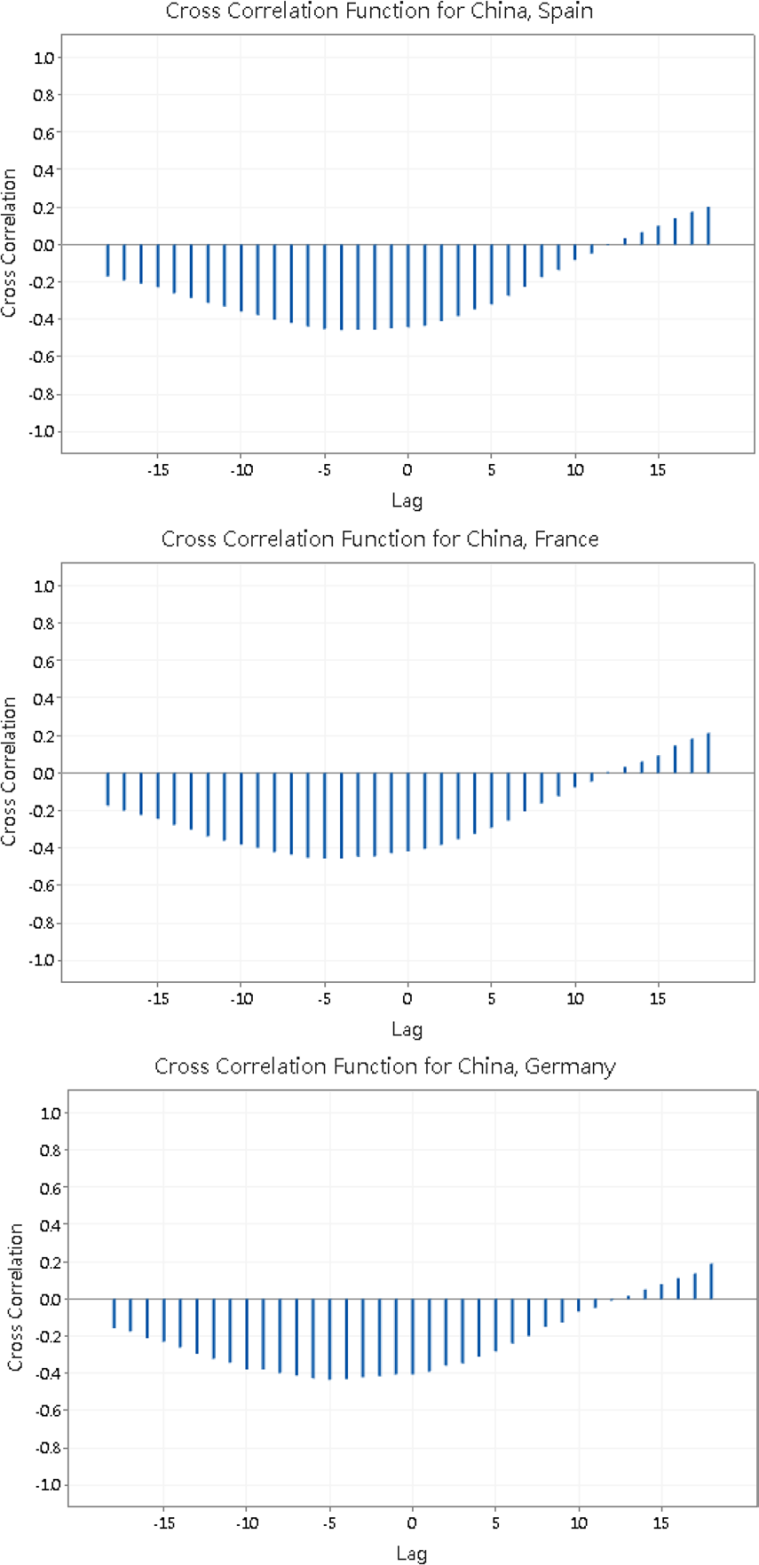
The cross correlation function between $\ln \left( {X_{i,t} + 1} \right)$ of China and other 7 economies

Then we examine the cross correlation function between $\ln \left( {X_{i,t} + 1} \right)$ of China and other 7 economies using the software Minitab 19:

Note that in each graph in Fig. 2, the x-axis “Lag” indicates the number of days of which $\ln \left( {X_{i,t} + 1} \right)$ of China leads $\ln \left( {X_{i,t} + 1} \right)$ of the other economy. The maximum number of lags that Minitab 19 can test is 18. From the figure, we can see that except Hong Kong, the cross correlation function between $\ln \left( {X_{i,t} + 1} \right)$ of China and other 6 economies follows a similar pattern: the cross correlation function is negative when the lag is negative or positive but small, but turns positive for large, positive lags. The cross correlation function attains a minimum when the lag is negative, i.e. the no. of confirmed cases of Covid-19 in China lags behind other economies if you treat them as having a negative correlation. This causes the abnormal result that the number of confirmed cases of Covid-19 in China is significantly Granger caused by other economies. Figure 2 shows that the cross correlation function turns positive and increases as the number of lag increases. This reveals that the no. of confirmed cases of Covid-19 in China in fact leads other economies by many days.

Table 4b displays the *p* values of the Granger causality test on $R_{i,t}$ of the 8 economies. Compared with Table 5, there are fewer entries of which the *p* values are smaller than 0.05. This indicates that at 5% significance level, the transmission of shocks between equity index return of the 8 economies is less significant than the transmission of shocks between the number of confirmed cases of Covid-19 of the 8 economies. One possible reason for this result is that the financial market is not solely affected by the Covid-19 outbreak. There are also other factors, e.g., the Sino-US trade war, affecting the financial market. Those factors may mitigate the effect of the Covid-19 outbreak, so the transmission of shocks becomes less significant. At 5% significance level, the equity index return of Italy significantly Granger causes the equity index return of 4 other economies, so we can identify Italy as a source of shock transmission between the 8 equity markets. Meanwhile, the equity index return of Japan is significantly Granger caused by the equity index return of 4 other economies, so we can identify Japan as a recipient of shock transmission between the 8 equity markets.

Table 4c shows the *p* values of the Granger causality test between $\ln \left( {X_{i,t} + 1} \right)$ and $R_{i,t}$ of each of the 8 economies. From the table, except for Hong Kong, the *p* value corresponding to $\ln \left( {X_{i,t} + 1} \right)$ -> $R_{i,t}$ is smaller than the p-value corresponding to $R_{i,t}$ -> $\ln \left( {X_{i,t} + 1} \right)$ for all other economies. This implies that for most economies, the no. of confirmed cases of Covid-19 Granger causes its equity index return rather than the reverse direction. At 5% significance level, the Granger causality test for $\ln \left( {X_{i,t} + 1} \right)$ -> $R_{i,t}$ is significant for Spain, France and Germany, but insignificant for Italy and all 4 East Asian economies. This shows that the transmission of shocks from the no. of confirmed cases of Covid-19 to the equity index return is more significant among the European countries than among the East Asian economies. This result coincides with the result of OLS regression that the slope coefficient is more significant for the European countries than for the East Asian economies (see Table 2). The Covid-19 outbreak has a more prevalent impact on the European financial markets than on the East Asian financial markets.

## Discussion and conclusion

In this study, we use OLS regression to test the impact of the Covid-19 outbreak on the equity index return of each individual economy during the early stage of the pandemic, and apply panel regression to examine the overall effect of the disease on the index return of the eight economies. We also add a new feature in this study: we conduct Granger causality test to examine the causality relationship. The following shows the main results:

1. The OLS regression model shows that at 5% significance level, the Covid-19 outbreak has a significant negative impact on the equity index return of all European countries and Japan, but the effect is insignificant on the equity index return of the remaining three East Asian economies.
2. The fixed effects model shows that the Covid-19 outbreak has a significant negative impact on the overall equity index return of the eight economies even at 0.1% significance level.
3. The Granger causality test shows that the transmission of shock is more significant between the no. of confirmed cases of Covid-19 in the 8 economies than between the equity index return of the 8 economies. Meanwhile, the transmission of shocks from the no. of confirmed cases of Covid-19 to the equity index return is more significant for the European countries than for the East Asian economies.

From the above results, we can see that the Covid-19 outbreak has an extremely significant negative impact on the overall equity index return of the eight economies. This shows that the disease is highly widespread. Many countries took quarantine measures, prohibiting people from going outdoors and closing many shops and restaurants. Hence their economic activities came to a standstill. The GDP growth of the U.S. fell to a record low of − 31.2% in the second quarter of 2020, while its unemployment rate rose to above 15% in the same quarter. Other major economies in the world also experienced a deep recession in year 2020, too. This reflects that the impact of the Covid-19 outbreak on the economy of the world is highly significant.

The individual OLS regression model reveals that the Covid-19 outbreak has a more significant impact on the European equity index return than on the East Asian equity index return. This reflects that the East Asian economies, having experience of dealing with the SARS outbreak in 2003, reacted faster in implementing quarantine measures to control the outbreak at a limited scale. In contrast, the European countries reacted slower, so the disease got out of control. The difference between Asian and western culture also contributes to this result. Most East Asian citizens do not think that freedom is the most important. They are willing to follow strict controls to stop disease spreading. However, most western citizens overemphasize freedom and liberty. They are reluctant to obey the preventive measures. This has three important implications. Firstly, when policy makers face a crisis that may potentially affect their economy, they have to react fast to mitigate the impact of the crisis. Otherwise, investors will lose confidence on the government in dealing with the crisis. They will hence sell their stocks, leading to a fall in the equity market. The impact of the crisis on the economy may be more significant, too. Secondly, investors should also be aware of an outbreak of disease or other risks and adjust their investments accordingly. For example, the Covid-19 outbreak affected the western economies more severe than the East Asian economies, so investors should avoid equities of western economies during the pandemic. Furthermore, the difference between Asian and western culture caused East Asian countries to be more successful in controlling the Covid-19 outbreak than their western counterparts. Hence western countries suffered a much deeper economic recession than their East Asian counterparts did. This changes many people’s view of the world that the western culture, which stresses on freedom and democracy, is better than the eastern culture. The East Asian countries have a greater potential of economic growth in the future than the western countries do, so it is more worthy to invest in East Asia. The Covid-19 outbreak results in a shift of power from the west to the east.

There are some limitations of this study. The first limitation is the accuracy of the data of number of confirmed cases of Covid-19 of the economies. Due to limited resources, every economy can only conduct Covid-19 tests on a proportion of all patients. Those patients who do not undergo Covid-19 tests may still contract the virus. Therefore, the actual number of confirmed cases of Covid-19 of the economies is larger than the figure announced by the government. Moreover, some people contracting Covid-19 do not have any symptoms. Mainland China did not include those patients who tested positive for Covid-19, but did not have any symptoms in its confirmed cases until 1 April 2020. This also makes the official number of confirmed cases of Covid-19 in mainland China smaller than the actual figure. Secondly, there are no control variables in our model. We do not include control variables because finding consistent controls across global markets in this era in which everything keeps on changing is difficult, especially when we have emerging markets like China in this study. Furthermore, Hünermund and Louw (2020) argue that the estimated effect sizes of control variables are unlikely to have a causal interpretation themselves. They recommend to refrain from reporting marginal effects of controls in regression tables and instead to focus exclusively on the variables of interest.
